# School Social Capital Mediates Associations Between ASD Traits and Depression Among Adolescents in General Population

**DOI:** 10.1007/s10803-022-05687-9

**Published:** 2022-08-02

**Authors:** Hiroyuki Mori, Tomoya Hirota, Rei Monden, Michio Takahashi, Masaki Adachi, Kazuhiko Nakamura

**Affiliations:** 1 Department of Psychology, Faculty of Health and Medical Science, Teikyo Heisei University, 2-51-4, Higashiikebukuro, Toshima-ku, Tokyo 171-0014 Japan; 2 Research Center for Child Mental Development, Graduate School of Medicine, Hirosaki University, 5, Zaifu, Hirosaki, Aomori, 036-8562 Japan; 3 Department of Neuropsychiatry, Graduate School of Medicine, Hirosaki University, 5, Zaifu, Hirosaki, Aomori 036-8562 Japan; 4 Department of Psychiatry and Behavioral Sciences, Weill Institute for Neurosciences, University of California San Francisco, San Francisco, CA USA; 5 Graduate School of Advanced Science and Engineering, Hiroshima University, 1-4-1 Kagamiyama, Higashi-Hiroshima City, Hiroshima Japan; 6 Smart-Aging Research Center, Tohoku University, 4-1 Seiryo-Machi, Aoba-ku, Sendai, 980-8575 Japan; 7 Faculty of Psychology, Meiji Gakuin University, 1-2-37 Shirokanedai, Minato-ku, Tokyo, 108‐8636 Japan

**Keywords:** Autism spectrum disorder, Adolescents, School social capital, Depression, Mediation, Moderation

## Abstract

Though autism spectrum disorder (ASD) traits are associated with depression, it is unclear if school social capital mediates their association. We examined whether school social capital mediates the association between ASD traits and depression, and moderation effect of sex on the mediation effect among adolescents in a general population sample (1750 males, 1779 females; equivalent 12–15 years old). The results of this study indicate that ASD traits are associated with depression among adolescents, and that this association is partly mediated by school social capital. Furthermore, the results of the moderated mediation analysis suggest that lower level of school social capital can lead to more increase level of depression for females than for males.

## Introduction

Autism spectrum disorder (ASD) is one of the neurodevelopmental disorders. Individuals with ASD are more likely to have co-occurring mental health problems compared to typically developing individuals (Lundström et al., 2015; Maskey et al., 2013; Simonoff et al., 2008, 2013). One study reported that approximately 70% of school-age children between 10 and 14 with ASD had at least one co-occurring psychiatric disorder (Simonoff et al., 2008). Autistic-like behaviors were referred to by family members of children with autism as broader autism phenotype (BAP) (e.g., Bailey et al., 1998; Piven et al., 1997). Then, a considerable amount of research has suggested that ASD traits are a continuum (Bölte et al., 2011; Constantino & Todd, 2003; Skuse et al., 2009) and that ASD traits exist in a general population sample (e.g., Constantino & Charman, 2016; Constantino & Todd, 2003; Kamio et al., 2013). Similar to individuals with ASD, ones with higher ASD traits in a general population are strongly associated with co-occurring psychiatric disorders and psychopathology (Hallett et al., 2010; Lundström et al., 2011; Saito et al., 2017). Among them, depression is one of the most serious psychiatric disorders because it is the most prevalent risk factor for suicide (Hawton et al., 2013). However, the mechanisms that explain the association between ASD traits and depression are poorly understood.

Recent research suggest that the association between ASD traits with depression is mediated through other factors. In adults with ASD, emotion regulation (Sáez-Suanes et al., 2020) and loneliness (Schiltz et al., 2021) were identified as mediators of the association between ASD traits and depression. In another study using British longitudinal data found that ASD traits at age 10 were associated with later depression at age 18 and that social communication impairment, including difficulties in awareness of others' feelings and reciprocal communication, had the strongest association with depression. Furthermore, the study revealed that children with social communication impairment were more likely to report being bullied and that bullying victimization mediated a substantial proportion of the variance of depression at age 18 between ASD traits and depression (Rai et al., 2018). In sum, these findings suggest that difficulties in social communication are associated with depression but that the association between social communication challenges and depression is partially accounted for by contextual factors such as bullying.

In recent years, there have been increasing reports of the involvement of social contexts, such as social relationship quality, and social support, in the association between ASD traits and depression (De-La-Iglesia & Olivar, 2015; Gotham et al., 2014; Hedley et al., 2018). A previous study in young adults in a general population sample reported that ASD traits were associated with mental health problems, including depression and that the association was partly mediated by the levels of social connectedness and loneliness (Stice & Lavner, 2019). These findings imply that contextual factors play an important role in the association between ASD traits and depression.

Social capital is a concept that encompasses the above-mentioned contextual factors, including relationships, a sense of belonging, and connectedness. Social capital is defined as “features of social organization, such as trust, norms, and networks, that can improve the efficiency of society by facilitating coordinated actions” (Putnam, 1993). A substantial number of studies have shown that social capital is associated with mental health (e.g., De Silva et al., 2005; Ehsan & De Silva, 2015; Fujiwara & Kawachi, 2008). For example, individuals who perceived a lower level of social capital were more likely to develop major depressive disorder than those who perceived a higher level of social capital (Fujiwara & Kawachi, 2008). A growing number of studies have revealed similar relationships between social capital and depression in children and adolescents (e.g., Bosacki et al., 2007; Ciairano et al., 2007; McPherson et al., 2014; Mori et al., 2022). Specifically, trust for friends (Bosacki et al., 2007), social cohesion (Aneshensel & Sucoff, 1996), supportive friendships (Ciairano et al., 2007), school cares, and dinner with parents (Fitzpatrick et al., 2005) are associated with depressive symptoms. Importantly, studies have reported the important roles of the social factors, including family, peer relationships, and school connectedness as risk factors for depression (Schulte-Körne, 2016; Shochet et al., 2006; Thapar et al., 2012).

With respect to the relationship between ASD traits in a general population and contextual factors, previous studies have shown that higher ASD traits are associated with poorer social interaction, more behavioral problems at school, less social connectedness, and lower sense of belonging (Hsiao et al., 2013; Jobe & White, 2007; Stice & Lavner, 2019; Whelan et al., 2021). While social capital was found to mediate the relationship between ASD traits and depression in young adults (Stice & Lavner, 2019), it is unclear if this mediation role of social capital can be extended to adolescence. Understanding this is critical given the findings from epidemiological studies reporting the onset of depression being in adolescence (Ghandour et al., 2019; Merikangas et al., 2010). Furthermore, consideration of school contextual factors is important given their contribution to the child’s social and psychological development (Eccles, 1999; Rutter, 1979). School contextual factors, including school connectedness, are identified to be contributing factors to their depression (Schulte-Körne, 2016; Shochet et al., 2006). Thus, clarifying the role of contextual factors, including school social capital, in adolescent mental health problems can lead to the development of appropriate interventions.

To this end, the present study aimed to clarify how school social capital could play a role for the relationship between ASD traits and depression among adolescents in a general population sample. We hypothesized that higher levels of ASD traits would be associated with more severe depression and lower levels of social capital. Previous studies have reported that depression in individuals with ASD was associated with the experience of bullying (Rai et al., 2018), IQ, coping with stress (DeFilippis, 2018), cognitive functioning (De-La-Iglesia & Olivar, 2015), emotion regulation (Sa´ez-Suanes et al., 2020), and loneliness (Schiltz et al., 2021). Given that there are several factors related to depression in individuals with ASD, we hypothesized that the association between ASD traits and depression could be partially mediated by school social capital, which is in line with a previous study by Stice and Lavner (2019). Therefore, the current study adopted a partial rather than a full mediation model. Importantly, previous studies have reported underlying sex differences in social behavior for typically developing individuals (Benenson & Christakos, 2003) as well as those with ASD (Head et al., 2014). The study in young adults (Stice & Lavner, 2019) reported that there was a significant indirect path from ASD traits to internalizing problems through social connectedness only for females. Thus, we also examined whether the student’s sex moderated the hypothesized mediation between ASD traits and depression by school social capital. Since little is known regarding the role of the student’s sex in the association between ASD traits and school social capital and depression, the second aim of the present study was exploratory, and thus we did not form a specific hypothesis corresponding to this aim.

## Methods

### Study Setting and Participants

In the present study, we used data from a community-based ongoing prospective cohort survey in national and public schools in Hirosaki city, Japan. The details of the study design are described elsewhere (Takahashi et al., 2018) and summarized below. The data have been collected annually since 2015. Current study involves data prospectively collected at 2016 (T1), 2017 (T2) and 2018 (T3). At each time point, we mailed letters containing information of the study to the parents/guardians of the students. Students whose parents/guardians did not consent to their participation in the study were excluded from the study. We distributed a set of questionnaires to all students via their schools, and students filled out the questionnaires in their classrooms. The classroom teachers explained the survey contents to the students and informed them that they were free to decide whether or not to participate in the study and that they could withdraw their consent at any time without any disadvantages, even after participation. We gathered information from various sources, including students, parents/guardians. The current study analyzed data collected from the students and their parents/guardians from T1 to T3. In total, 4235 students (grades 7–9th, equivalent to 12–15 years old at T3) and their parents/guardians were enrolled to the study.

### Measurements

#### School Social Capital

School social capital was measured at T3 using the “school trust and social cohesion” subscale of the Japanese version of the Social Capital Questionnaire for Adolescent Students (SCQ-AS; Paiva et al., 2014). It comprised of 12 items, which provided a quantitative measure of social capital, and the construct validity had been confirmed (Paiva et al., 2014). The reliability and factorial validity of the Japanese version have been confirmed (Hirota et al., 2019). This subscale consisted of 8 items scored on a 3-point scale (I agree = 3; I do not agree or disagree = 2; I disagree = 1). The subscale score ranged from 8 to 24, with a higher score indicating a higher level of school social capital.

#### ASD Traits

ASD traits were measured at T1 using the Autism Spectrum Screening Questionnaire (ASSQ; Ehlers et al., 1999). The ASSQ was administered to the parents/guardians of the participating students, only at T1. The ASSQ is a screening tool to identify ASD in school-aged children that consists of 27 items rated on a 3-point scale. Of the 27 items, 11 are on social interactions, 6 are on communication problems, and 5 are on restricted and repetitive behavior. The remaining items pertain to motor clumsiness and other associated symptoms. Psychometric properties of the Japanese version of the ASSQ were validated in a general population sample (Ito et al., 2014). The total score ranges from 0 to 54, with a higher score indicating higher ASD traits.

#### Depression

Depression was measured from T1 to T3 using a Japanese version of the Depression Self-Rating Scale for Children (DSRS-C; Birleson, 1981). The reliability and validity of the Japanese version have been confirmed (Murata et al., 1996). The DSRS-C consists of 18 items scored on a 3-point scale (never = 0; sometimes = 1; always = 2). The total score ranges from 0 to 36, with a higher score indicating that the child has more severe depression. Murata et al. (1996) suggested that the cut-off of the Japanese version should be ≥ 16 points. The data at T1 and T2 were included in the multiple imputation model as auxiliary variables.

#### Other Measures

Besides the above-mentioned measures, two other measures were used in the study: Quality of Life (QoL) collected from the students and behavioral/emotional problems collected from the parents/guardians. QoL was measured from T1 to T3 using a Japanese version (Kobayashi & Kamibeppu, 2010) of the Pediatric Quality of Life Inventory 4.0 Generic Scores (PedsQL; Varni et al., 1999). Behavioral/emotional problems were measured at each time point using a Japanese version (Matsuishi et al., 2008) of The Strengths and Difficulties Questionnaire (SDQ; Goodman, 1997). These two measures (PedsQL at T3 and SDQ from T1 to T3) are not the main interest of our study but they were included in the multiple imputation model as auxiliary variables.

### Statistical Analyses

All analyses were performed using R 4.1.0 (R Core Team, 2021). First, multiple imputation was performed followed by mediation analyses.

#### Missing Data

Students whose parents/guardians did not provide any data for the ASSQ were excluded from the study. Introducing too much bias in the multiple imputation procedure was avoided while retaining as much students as possible. This resulted in selecting 3529 (83.3%) of the initial 4235 students for the mediation analysis. Of these, 3339 students and their parents/guardians did not have any missing data on all the above-mentioned measures. In the remaining sample, 2.2% of the data was missing. These missing data were imputed 20 times, using the mice 3.13.0 package (Van Buuren & Groothuis-Oudshoorn, 2011). Besides the demographics (i.e., grade and sex), all the above-mentioned measures collected from the students and parents/guardians were included in the imputation model. Following the rule by Rubin (Rubin, 1987), mediation analysis was performed on each of the 20 imputed datasets and then combined to acquire the estimates.

#### Main Analyses

**Fig. 1:**
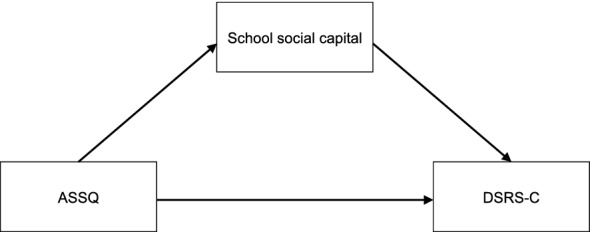
Model of school social capital as the mediator between ASD traits and depression

**Fig. 2:**
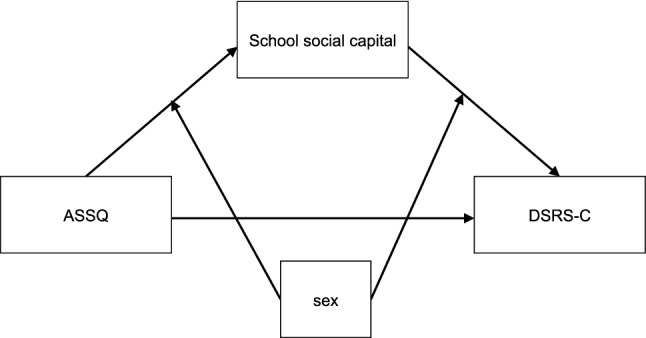
Model of sex as a moderator of the mediation model

First, mediation analysis, or structural equation modeling in a broader term, was performed to study whether the association between ASD traits and depression was mediated by school social capital (Fig. 1). Next, we conducted the moderated mediation analysis to examine whether the indirect effects of ASD traits on depression was moderated by sex (Fig. 2). The analysis was performed by using “sem” function in semTools (Jorgensen et al., 2021).

## Results

### Sample Characteristics

Sample characteristics of the participants are presented in Table 1. In general, the sample distributed equally to all grade and sex. Moreover, the sample characteristics were comparable between the overall sample and the analyzed sample. The school social capital scores were similar between sex, while the ASD traits scores was higher in male and the depression scores was higher in female. The correlations between school social capital, ASD traits and depression are presented in Table 2. As expected, there was a negative correlation between shool social capital and ASD traits [*r* =  − 0.18; 95% CI (− 0.22 to − 0.15), *p* < 0.001], between school social capital and depression (*r* =  − 0.66; 95% CI (− 0.68 to − 0.64), *p* < 0.001]. There was a positive correlation between ASD traits and depression [*r* = 0.20; 95% CI (0.16 to 0.23), *p* < 0.001].

**Table 1:** Sample characteristics

	Overall sample (N = 4235)		Analyzed sample (N = 3529)	
	Male	Females	Male	Females
Grade 7 (age 12–13)	695	675	567	581
8 (age 13–14)	679	694	549	582
9 (age 14–15)	768	724	634	616
	Mean (SD)		Mean (SD)	
School social capital^a^	18.9 (4.3)	19.0 (4.1)	19.0 (4.3)	19.1 (4.1)
ASD traits^b^	4.4 (5.6)	3.6 (4.5)	4.4 (5.6)	3.6 (4.5)
Depression^c^	8.4 (5.9)	9.5 (6.5)	8.2 (5.9)	9.4 (6.4)

^a^ “school trust and social cohesion” subscale score of the Japanese version of the Social Capital Questionnaire for Adolescent Students

^b^Autism Spectrum Screening Questionnaire score

^c^Depression Self-Rating Scale for Children score

**Table 2:** Correlations among all variables

	1	2	3
1. School social capital^a^		− 0.193***	− 0.674***
2. ASD traits^b^	− 0.181***		0.192***
3. Depresion^c^	− 0.661***	0.218***	

Pearson correlation coefficients for males are presented below the diagonal; coefficients for females are presented above the diagonal

^a^ “school trust and social cohesion” subscale score of the Japanese version of the Social Capital Questionnaire for Adolescent Students

^b^Autism Spectrum Screening Questionnaire score

^c^Depression Self-Rating Scale for Children score

***p < 0.001

#### Mediation Analysis

**Fig. 3:**
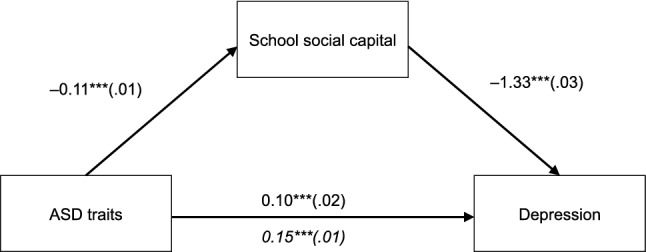
Mediation model of indirect of ASD traits on depression thorough school social capital. Standardized β coefficients between variables are displayed (with standard errors in brackets). Indirect effect is presented as italicized ***p < 0.001

The path coefficients of the mediation analysis are presented in Fig. 3. In short, the ASD trait was positively associated with depression (β = 0.10) and negatively associated with social capital (β =  − 0.11). Furthermore, school social capital was negatively associated with depression (β =  − 1.33). The magnitude of the indirect effect of ASD traits on depression mediated by school social capital was 0.15, represented by the beta coefficient, indicating that school social capital acted as a mediator between ASD traits and depression. Note that all the estimated coefficients were statistically significant (*p* < 0.001).

#### Moderated Mediation Analysis

**Fig. 4:**
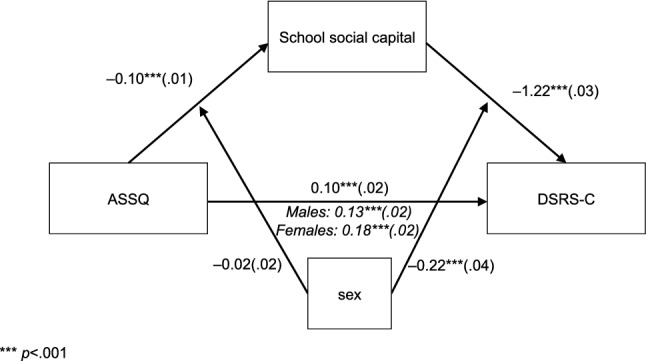
Moderated mediation model of sex as moderator. Standardized β coefficients between variables are displayed (with standard errors in brackets). Indirect effects thorough school social capital are presented as italicized ***p < 0.001

**Fig. 5:**
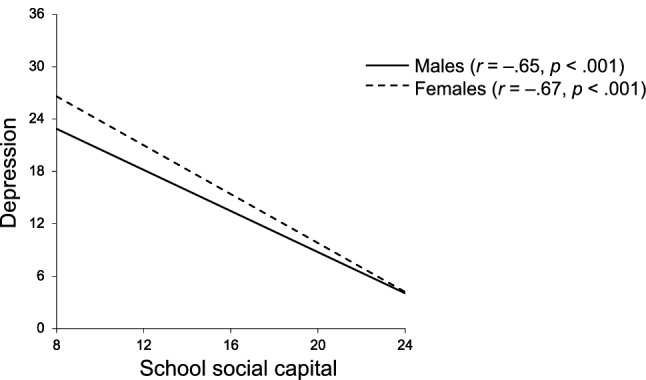
Moderating role of sex on the association between ASD traits and depression. Depression, Depression Self-Rating Scale for Children score; school social capital, “school trust and social cohesion” subscale score of the Japanese version of the Social Capital Questionnaire for Adolescent Students

The path coefficients of the moderated mediation analysis are presented in Fig. 4. Indirect effects of ASD traits on depression mediated by school social capital were significant for both sexes. The indirect effect of ASD traits on depression, mediated by school social capital, was slightly stronger in females (β = 0.18, *p* < 0.001) than in males (β = 0.13, *p* < 0.001). The interaction between ASD traits and sex was not significantly associated with school social capital (β =  − 0.02, *p* = 0.263). Whereas, the interaction between school social capital and sex was significantly associated with depression (β =  − 0.22, *p* < 0.001). Simple slope analysis revealed that path from school social capital to depression was slightly stronger for females (β =  − 1.44, *p* <0 .001) than for males (β =  − 1.22, *p* < 0.001). To understand the pattern of this moderation effect, the relation between school social capital and depression is plotted by the moderator in Fig. 5, for each sex.

## Discussion

The present study aimed to clarify the role of school social capital in the association between ASD traits and depression in a general population sample of adolescent students in Hirosaki city, Japan. The results in this study supported our hypothesis that those with higher ASD traits reported the lower level of school social capital and more severe level of depression and that the association between ASD traits and depression was partially mediated by school social capital.

To the best of our knowledge, we are the first to report that school social capital is associated with ASD traits in adolescents. Our findings are in line with previous research that reported the association of ASD traits with social capital components, such as social relationships (Jobe & White, 2007), connectedness (Stice & Lavner, 2019), and belonging (Pelton & Cassidy, 2017). However, targeted populations were adults, and social capital indicators were measured more broadly in the above-referenced studies. With respect to children and adolescents, previous research reported that adolescents with ASD perceived lower school connectedness than typically developing adolescents (Hebron, 2018) and that individuals with higher ASD traits in a general population was strongly associated with poor peer relationships (Hsiao et al., 2013). This may be explained by deficits in theory of mind (Baron-cohen et al., 1985), one of the core features of ASD, leading to challenges with awareness and understanding of the feelings and mental states of the others. Due to deficits in theory of mind, those with higher ASD traits may have difficulty constructing reciprocal friendships and mutual aid, one of the components of social capital (Bauminger et al., 2008). Consequently, those with higher ASD traits may perceive lower level of school social capital.

Our results also showed that adolescents with higher ASD traits have more severe depression. The results are consistent with previous studies that investigated the relationship between ASD traits and depression in the general population (Hallett et al., 2010; Rai et al., 2018).

Furthermore, we also revealed that the association between ASD traits and depression was partially mediated by school social capital. Our results could extend the findings in previous studies that social contexts can explain the association between ASD traits and depression (De-La-Iglesia & Olivar, 2015; Gotham et al., 2014; Hedley et al., 2018; Stice & Lavner, 2019) to adolescents in a general population sample. As the research in young adults reported that connectedness that was one of social capital played an important role in the association ASD traits and internalizing problem (Stice & Lavner, 2019). Our results indicate school social capital could similarly account for the association between ASD traits and depression among adolescents in the general population and suggest that the school context be taken into account in the increase of depression in adolescents with ASD traits, especially challenges with social communication and interactions.

School is a location where children and adolescents spend a great amount of time, with a combination of social experiences and challenges, learning demands and mental and emotional stress (Rutter, 1979). Students with a positive school context, including school connectedness, are associated with lower level of depression (Schulte-Körne, 2016; Shochet et al., 2006) and suicidality (Young et al., 2011). This suggests that positive sense of school context can protect against mental health problems. Students with ASD experience more loneliness than typically developed students, and have low social network status (Locke et al., 2010) and few reciprocal friendships (Kasari et al., 2011). Even when ASD traits are below the clinical threshold, peer problems and teacher-student interactions are impaired (Hsiao et al., 2013). Therefore, promoting school social capital, including school connectedness, for adolescents in a general population sample is reasonable and beneficial especially for those who are vulnerable to mental health problems (e. g. those with ASD traits; Cumming et al., 2017).

We also examined whether the mediation effect of school social capital on the relationship between ASD traits and depression differed by the student’s sex. The moderated mediation analysis revealed that the association between ASD traits and school social capital was not moderated by sex. The previous study in children and adolescents in the general population reported that the association between ASD traits and problems with peers was moderated by sex such that it was particularly stronger in females (Hsiao et al., 2013). The peer relationships can be included by components of school social capital, but the differences of concepts between them may have influenced discrepancies. The moderation effect on association between ASD traits and school social capital, including school connectedness and peer relationships, needs to be further examined.

The moderated mediation analysis also revealed that indirect effects of ASD traits on depression mediated by school social capital were significant for both sexes. The study in young adults (Stice & Lavner, 2019) reported that there was a significant indirect path from ASD traits to internalizing problems through social connectedness only for females. This inconsistent finding may be explained by differences in outcome variables. One study suggest that school connectedness predicted depressive symptoms for both sexes but predicted anxiety for girls only in adolescent (Shochet et al., 2006). Stice and Lavner (2019) measured internalizing symptoms including anxiety and depression, which may have led to sex differences. In addition, the result indicates that sex moderated the association between school social capital and depression, although differences in the magnitude of the coefficients were slight. This suggested that lower level of school context can lead to more increase level of depression for females than for males in the general population. The previous studies in the general population showed that females had higher quality, closer friendships (e.g., Camarena et al., 1990; Rose & Asher, 1999) and felt more distress than males about the potential termination of their current closest same-sex friendship (Benenson & Christakos, 2003) and that females were more sensitive to contextual influences on peer relationships than are males (Hardy et al., 2002). Moreover, in studies of clinical samples, girls with ASD recognized the importance of close friends for emotional and social support but that having friends was simultaneously hard work (Sedgewick et al., 2019). Future research on sex differences in social context regarding the association between ASD traits and mental health problems should be conducted.

### Implications

The results of present study indicate that ASD traits are associated with depression among adolescents and that this relation is partly mediated by levels of school social capital. This suggests that interventions focused on school social capital may prevent worsening mental health problems in students with higher ASD traits. For example, one study showed that lower school participation was associated with greater anxiety in adolescents with ASD who attended mainstream schools. (Chang et al., 2019). These activities and participation do not necessarily involve interaction with others but do represent some form of engagement with one’s external world and can lead to connectedness or sense of belonging. Future research should examine the relationship between the mental health of adolescents with ASD traits and not only their individual perceived social capital (cognitive social capital) but also their actual participation in activities (structural social capital), such as school participation.

### Strengths and Limitations

This study has a number of strengths; to the best of our knowledge, it was the first to clarify the mediating role of school social capital in understanding the association between ASD traits and depression. The use of Structural equation modeling (SEM) was also a notable strength of the study. SEM allowed the simultaneous estimations of direct and indirect effects. Another strength of this study is the use of data based on a large community sample with a high retention rate of participants which provided a mostly unbiased adolescent sample of age cohorts. In addition, we used multiple imputation to retain as much sample as possible without introducing too much bias as well as evaluate the uncertainty of the estimations.

The study has several limitations. First, as school social capital and depression were collected at the same time point, the study was unable to show causal mediating effect of school social capital between ASD traits and depression. Second, we excluded participants without ASSQ data (13.3%) to minimize introducing biases in the imputation procedure; however, this could lead to a potential selection bias. Third, we use only SCQ-AS to measure school social capital, which was specific to the quantity of perceived social capital. Assessing the impact of social capital in other domains will be important extension of the current study. Fourth, the identified mediators of the association between ASD traits and depression, such as emotion regulation (Sáez-Suanes et al., 2020), loneliness (Schiltz et al., 2021), and being bullied (Rai et al., 2018), were not obtained in this study. Fifth, we collected data on only grade and sex, even though other demographic factors could also affect the model examined in the present study. For example, socioeconomic status (Goodman, 1999) is associated with adolescent mental health. Finally, because our study was limited to a specific region of Japan and did not consider social, contextual, and cultural factors, we could not examine whether the data in this study was representative of Japanese adolescents and how cultural factors can affect social capital and other constructs. To replicate our findings, multi-region studies should be conducted to determine the role of school social capital in the association between ASD traits and depression.

## Conclusion

The current results indicate that ASD traits are associated with depression among adolescents and that this association is partly mediated by level of school social capital. Moreover, the results of moderated mediation analysis suggest that lower level of school social capital can lead to more increase level of depression for females with ASD traits than for males with ASD traits in the general population. Overall, these findings point to the importance of continuing to study the contextual factors of those with ASD traits, while also identifying other relevant factors associated with mental health in order to develop more effective interventions.
